# Periosteal Chondroma of the Pelvis: An Uncommon Tumor in an Unusual Location

**DOI:** 10.7759/cureus.17163

**Published:** 2021-08-13

**Authors:** Gautham Prabhakar, Ameesh Dev, Fatemeh Ghazanfari Amlashi, Rajiv Rajani

**Affiliations:** 1 Orthopaedic Surgery, UT Health San Antonio, San Antonio, USA; 2 Pathology, UT Health San Antonio, San Antonio, USA; 3 Orthopaedic Oncology, UT Health San Antonio, San Antonio, USA

**Keywords:** periosteal chondroma, orthopedic tumor, pelvis

## Abstract

Periosteal chondromas (PCs) are rare tumors composed of hyaline cartilage that are typically present in long bones and tubular bones of the hand. These lesions are easily mistaken for other, more common tumors. This study reports a case of PC located in the posterior pelvis of a 24-year-old female. The patient initially presented with a four-month history of pelvic pain with a presumptive diagnosis of endometriosis. However, when an MRI was performed, a 6.0 cm x 5.6 cm x 4.5 cm mass was found along the right posterior ilium extending to the ipsilateral sacroiliac joint. The patient underwent intralesional excision and curettage of the mass. Histologic analysis of the excised lesion revealed a proliferation of chondrocytes and abundant hyaline cartilage without chondroblasts, further suggesting the diagnosis of PC. The current study highlights the unusual location of this rare tumor and alerts the physician of the clinical presentation and differential diagnosis.

## Introduction

Periosteal chondroma (PC) is an uncommon, benign cartilaginous tumor that typically arises under or within the periosteum of cortical bone [1,2]. They affect both adults and children, however; it has been found to occur mostly in patients less than 30 years of age [2]. PC is usually less than 3 cm and has a tendency to affect the metaphyses of long bones, particularly the proximal humerus, distal femur, and upper extremity phalanges [1-4]. The most common symptom of this pathology is pain; however, many cases are found incidentally [5]. Radiographically, PC appears as a cortically situated, lobulated soft tissue mass with erosion, also known as cortical saucerization. There may also be medullary sclerosis, periostitis, and cortical buttressing on X-ray [4]. Diagnosis is further supported with histopathological features such as the prominence of hyaline cartilage, binucleation, and nuclear pleomorphism [1-4]. To the authors’ knowledge, there has been no report of PC in the ilium or sacroiliac joint. This study reports a unique and interesting presentation of PC in the pelvis of a 24-year-old female.

## Case presentation

**Figure 1:**
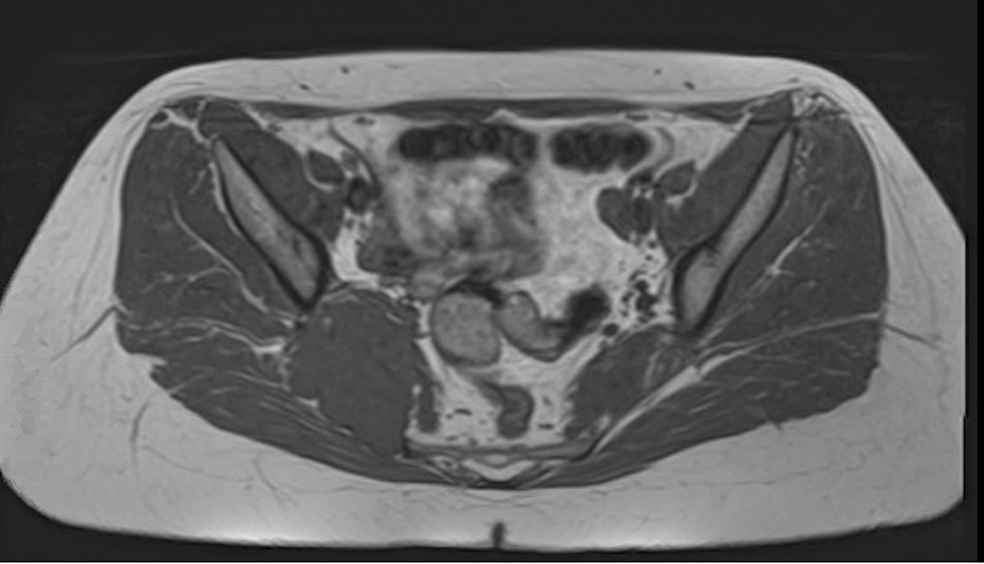
Axial view of T1. MRI showing 6 cm x 5.6 cm x 4.5 cm mass along the right ilium extending into the ipsilateral sacroiliac joint.

**Figure 2:**
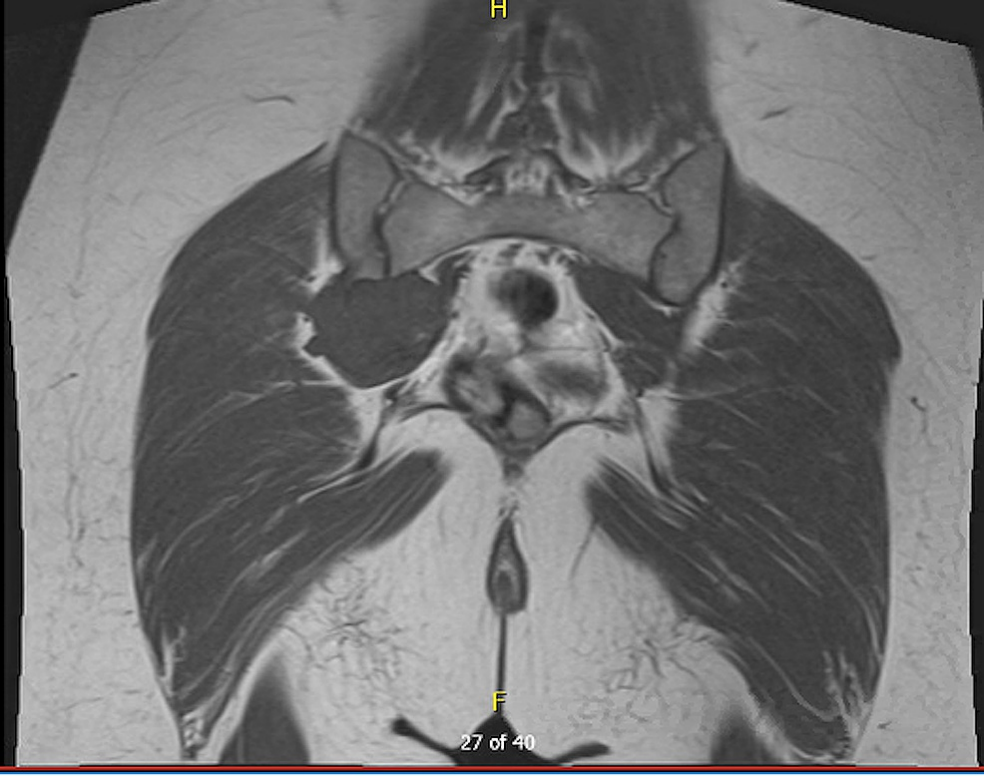
Coronal view of T1. MRI showing 6 cm x 5.6 cm x 4.5 cm mass along the right ilium extending into the ipsilateral sacroiliac joint. MRI showing 6 cm x 5.6 cm x 4.5 cm mass along the right ilium extending into the ipsilateral sacroiliac joint.

A 24-year-old female with polycystic ovarian syndrome presented with a four-month history of gradually worsening pelvic discomfort and pain, fatigue, weakness in gait, and sciatica-like symptoms. She initially presented to our level one academic trauma center for several days of urinary incontinence. On the current presentation, she stated that she was being worked up for endometriosis and was pending an MRI and diagnostic laparoscopy. She denied any inciting trauma or similar episodes in the past. Due to increasing pelvic pain and incontinence, an MRI was performed revealing a 6.0 cm x 5.6 cm x 4.5 cm mass found along the right ilium extending to the ipsilateral sacroiliac joint (Figures 1-2). Interventional radiology performed a CT-guided biopsy which showed a low-grade cartilaginous lesion. The patient was referred to the Orthopedic Oncology Clinic and the decision was made to take her to surgery for an intralesional curettage and excision of the right pelvic PC.

**Figure 3:**
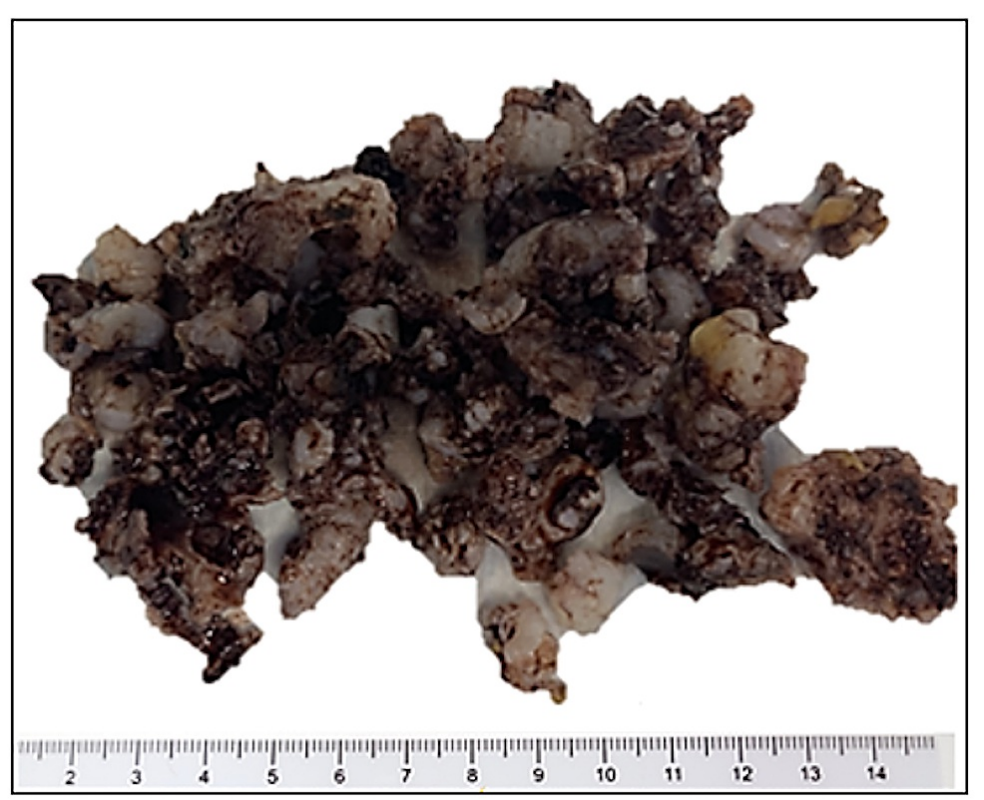
Gross picture of periosteal chondroma with multiple gray to tan brown soft tissue.

**Figure 4:**
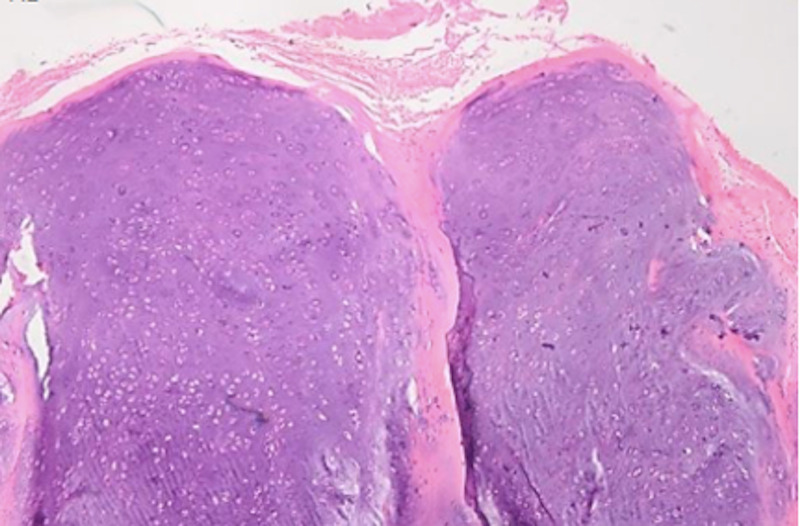
Discrete lobulated tumor consisting of moderately cellular hyaline cartilage (x10).

**Figure 5:**
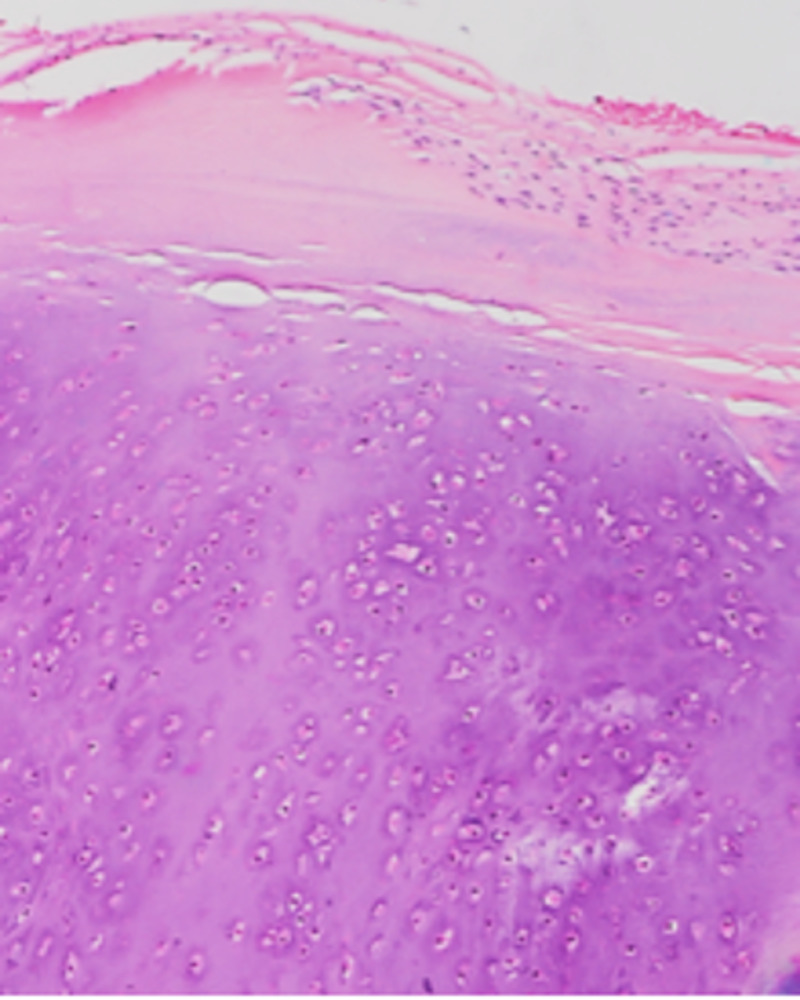
Bland chondrocytes and overlying periosteum (x20).

A 6 cm longitudinal incision was made in the direction of the gluteus maximus muscle fibers. The muscle fibers of the gluteus maximus were split longitudinally so that the upper 1/3 and lower 2/3 were separated to preserve the blood supply. The 6 x 5 cm mass was appreciated on the right inferior ilium extending to the sacral ala and sacroiliac joint. The mass was a well-circumscribed, multilobulated, low-grade cartilage appearing neoplasm (Figure 3). Fortunately, there was no destruction or significant invasion of the surrounding bone to warrant use of bone graft or cement. Intralesional curettage was performed with the use of curettes and rongeurs. Additionally, argon laser was utilized as an adjuvant to extend margins. Histopathologic analysis of the excised lesion revealed a discrete lobulated tumor consisting of hypo- to moderately cellular hyaline cartilage with bland neoplastic chondrocytes residing in lacunar spaces (Figures 4-5).

The patient had an unremarkable neurovascular exam of the right lower extremity postoperatively and the pain was well controlled. At the follow-up visit six weeks later, the patient reported significant improvement in her pelvic pain. There were no neurologic deficits, weakness, or instability of the right lower extremity.

## Discussion

PCs are rare hyaline cartilage tumors that typically account for less than 2% of all chondromas [2]. It is a slow-growing lesion that predominantly affects patients between 10 and 30 years. Reports in the literature describe PC being found in the proximal humerus, distal femur, and tubular bones of the hand [1-2]. The patient in this study presented with a PC on the posterior pelvis adjacent to the sacroiliac joint. Other unusual cases of PC have been documented in the cervical spine, clavicle, ischium, and ribs [6-9]. Optimal management of PC is wide resection due to local destruction and possible recurrence. One study reviewed 165 cases of PC and reported a recurrence rate of 3.6% [10].

The pathogenesis of PC is poorly understood. However, similar to enchondroma, PC frequently harbors heterozygous mutations in isocitrate dehydrogenase (IDH) genes mostly involving IDH1 mutation at R132 [11-13]. In addition, cytogenetic studies of PC have shown a loss of chromosome 6 and rearrangements of 2q37, 4q21-25, and 11q13-15 [14]. Panagopoulos et al. also reported chromosome aberrations of 12q13-15 in two of their four cases [12]. In existing literature, there has been no association shown between PC and other tumors or family history of cancer. Of note, this patient did have a family history significant for treatment-responsive breast cancer in her mother. 

The three fundamental radiographic features of PC are: scalloping/remodeling of the adjacent bony cortex, presence of cartilaginous matrix radiographically, and possible soft tissue mass component [15]. On MRI, a PC typically appears as a well-circumscribed, juxtacortical mass with an intermediate signal intensity on T1-weighted images and high-signal intensity on T2-weighted images [3,16]. Extraosseous soft tissue edema may be present adjacent to the lesion on T2-weighted imaging [3]. The imaging features in the current study are consistent with characteristics of PC found in the literature. In this case, the tumor was a 6 cm x 5.6 cm x 4.5 cm with high heterogeneity on T2 and low heterogeneity on T1. 

On histology, PC may show hypercellularity, nuclear enlargement, binucleation, and myxoid changes, which may resemble low-grade chondrosarcoma [1]. PCs are typically well circumscribed and demarcated from the underlying cortex. Similar to enchondroma, the hyaline matrix can be hypo- to moderately cellular and neoplastic chondrocytes are typically bland. Increased cellularity and mild cytological atypia may occasionally be seen but infiltrative growth into surrounding soft tissue or permeation into underlying bone is absent [11]. PC should be differentiated from chondrosarcoma, and parosteal and periosteal osteosarcoma. Periosteal chondrosarcomas are larger in average and are associated with cortical or soft tissue invasion [11]. Parosteal osteosarcoma shows neoplastic bone formation with an appearance of intermediate grade osteosarcoma intermixed with cartilaginous elements. Parosteal osteosarcoma may contain a cartilage cap, but the arrangement of chondrocytes tends to be more disorganized and the underlying bony trabeculae exhibit a characteristic parallel architecture with intervening bland spindle cells. Additionally, the hallmark is bone formation without osteoblastic rimming [11]. As PC is a benign tumor, there are typically no mitotic figures, significant atypia, or necrosis found on histology [17]. 

This patient also had an unusual feature of incontinence and sciatica-like symptoms, including numbness and weakness of the right lower extremity. There have been no documented cases of PC presenting with incontinence, however, other primary bone tumors have been noted to cause incontinence due to the location near the sacrum [18]. De Moraes FB et al. report a similar case in a 42-year-old female with an osteochondroma of the left ischium who suffered from progressive right hip pain, lower limb paresthesias, and sciatica-like pain. The patient in the study experienced resolution of symptoms following en-bloc resection of the tumor with no recurrence after a two-year follow-up [19]. Our patient likely experienced incontinence and sciatica due to the tumor’s unique location along the sacroiliac joint and close proximity to the sacral plexus.

Although surgical resection is recommended for PC, we chose to perform an intralesional curettage with the use of adjuvants. This was due to the significant morbidity that would be associated with this area with a wide resection. Although this likely increases the risk of local recurrence, the authors felt that an intralesional procedure would prevent the potential loss of neurologic function and bony stability.

## Conclusions

This case elucidates radiographic and histological features suggestive of PC that occurred in an unusual pattern along the right ilium extending into the ipsilateral sacroiliac joint. This unique location caused significant pelvic pain, and the patient was delayed proper treatment due to a presumptive diagnosis of endometriosis. In summary, the physician should remember this rare tumor and include PC of the pelvis in the differential diagnosis when presented with pelvic pain, altered gait, muscle weakness, and sciatica-like symptoms.
